# Bronchopulmonary Dysplasia Starts in Utero: MicroRNA Dysregulation Across the Fetal–Neonatal Continuum in the Bronchopulmonary Dysplasia Development

**DOI:** 10.1111/jcmm.71427

**Published:** 2026-11-02

**Authors:** Serafina Perrone, Silvia Carloni, Virginia Beretta, Vincenzo Raitano, Pietro Leuratti, Luca Buttarelli, Elisa Caselli, Sofia Gerevini, Maria Cristina Albertini, Serena Benedetti, Francesco Nonnis‐Marzano, Gianfranco Maffei, Eloisa Gitto, Giovanni Vento

**Affiliations:** ^1^ Neonatology Unit, Department of Medicine and Surgery University of Parma, Pietro Barilla Children's Hospital Parma Italy; ^2^ Department of Biomolecular Sciences University of Urbino Carlo Bo Urbino Italy; ^3^ Department of Chemistry, Life Sciences and Environmental Sustainability University of Parma Parma Italy; ^4^ Neonatology and Intensive Care Unit Policlinico Foggia Hospital Foggia Italy; ^5^ Neonatal and Pediatric Intensive Care Unit, Department of Human Pathology of the Adult and Developmental Age “Gaetano Barresi” University of Messina Messina Italy; ^6^ Neonatology Unit Fondazione Policlinico Universitario “Agostino Gemelli” IRCCS Rome Italy

**Keywords:** angiogenesis, bronchopulmonary dysplasia, fetal growth restriction, microRNA, oxidative stress, prematurity

## Abstract

Bronchopulmonary dysplasia (BPD) is a heterogeneous developmental lung disorder resulting from the interaction between antenatal susceptibility and postnatal injury. Increasing evidence suggests that its biological origins may precede preterm birth, with placental dysfunction, fetal growth restriction, inflammation and oxidative stress influencing pulmonary developmental trajectories before exposure to the extrauterine environment. MicroRNAs (miRNAs), small non‐coding RNAs that regulate gene expression at the post‐transcriptional level, are emerging as potential molecular mediators linking these fetal and neonatal processes. This educational review examines miRNA dysregulation across the fetal–neonatal continuum of BPD. We first discuss the role of miRNAs in physiological lung development and their disruption following premature birth. We then explore how placental dysfunction and fetal growth restriction may influence miRNA networks, including placenta‐enriched C19MC miRNAs, thereby contributing to antenatal susceptibility to BPD. Particular attention is given to miRNA‐mediated regulation of pulmonary angiogenesis and VEGF signaling and to the bidirectional relationship between miRNAs and oxidative stress, including modulation of NRF2‐dependent antioxidant responses. Beyond their mechanistic relevance, miRNAs offer potential translational opportunities. Their stability and detectability across different biological compartments—including placenta, cord blood, peripheral blood, respiratory samples and extracellular vesicles—make them attractive candidates for longitudinal biomarker profiling, while experimental modulation using miRNA mimics and inhibitors provides proof of concept for future therapeutic approaches. However, heterogeneity in biological sampling, analytical methods, developmental timing and BPD phenotypes, together with challenges in tissue‐specific delivery and off‐target effects, currently limit clinical translation. Rather than identifying a single ‘BPD miRNA’, future research should focus on dynamic, multi‐compartment miRNA networks integrating antenatal programming with postnatal exposures. Such an approach may improve molecular endotyping, early risk stratification and ultimately the development of individualized preventive and therapeutic strategies for BPD.

## 1 Introduction

Bronchopulmonary dysplasia (BPD) remains the most common chronic respiratory complication of extreme prematurity and continues to affect a substantial proportion of very preterm infants despite major advances in perinatal care and respiratory management. Current estimates indicate that BPD develops in up to 45% of infants born before 29 weeks of gestation, with considerable variability according to gestational age, clinical characteristics and diagnostic criteria [1, 2]. Beyond the neonatal period, BPD is associated with persistent abnormalities in lung function, recurrent respiratory morbidity, pulmonary vascular disease and adverse neurodevelopmental outcomes, highlighting its lifelong clinical relevance [3, 4].

The definition of BPD has evolved substantially to reflect changes in neonatal respiratory care and the heterogeneous clinical spectrum of the disease. Contemporary definitions primarily rely on the type and intensity of respiratory support required at 36 weeks' postmenstrual age (PMA). In particular, Jensen et al. proposed a three‐grade classification based on respiratory support at 36 weeks' PMA, which showed improved prediction of subsequent respiratory morbidity compared with previous oxygen‐based definitions [2, 5, 6]. Nevertheless, these classifications identify BPD when chronic lung disease is already established and provide limited insight into the biological processes operating during earlier stages of disease development.

BPD is increasingly recognized as a developmental disorder resulting from the interaction between an immature lung and multiple antenatal and postnatal insults. Antenatal conditions, including chorioamnionitis, premature rupture of membranes, hypertensive disorders of pregnancy and fetal growth restriction, may alter the intrauterine environment and influence lung development before birth [7, 8]. Following preterm delivery, extreme immaturity, mechanical ventilation, supplemental oxygen exposure, respiratory distress syndrome, patent ductus arteriosus, sepsis, blood transfusions and microbiota dysbiosis provide additional inflammatory and oxidative challenges [9, 10, 11]. Rather than acting independently, these exposures converge on interconnected biological pathways involving inflammation, oxidative stress, vascular dysfunction and impaired tissue repair, ultimately disrupting normal alveolar and pulmonary vascular development [12, 13, 14].

Oxidative stress represents a particularly important component of this pathogenic network. The transition from the relatively hypoxic intrauterine environment to extrauterine life exposes the immature lung to a marked increase in oxygen tension at a time when antioxidant defenses are still underdeveloped [15, 16]. Supplemental oxygen and mechanical ventilation can further amplify reactive oxygen species (ROS) generation and inflammatory cell recruitment. Excessive ROS production, in the presence of insufficient antioxidant capacity, promotes lipid, protein and DNA oxidation and may induce apoptosis or dysfunction of alveolar epithelial and pulmonary endothelial cells. Alveolar epithelial Type II cells are particularly relevant because of their roles in surfactant production, epithelial repair and maintenance of alveolar homeostasis. Their injury, together with disruption of pulmonary angiogenesis, contributes to the simplified alveolar architecture and abnormal vascular development characteristic of contemporary BPD [10, 17].

However, the biological response to perinatal injury is not determined solely by the magnitude of the insult. Increasing evidence indicates that epigenetic and post‐transcriptional mechanisms regulate how the developing lung responds to inflammation, oxidative stress, hypoxia, hyperoxia and altered angiogenic signaling. Among these mechanisms, microRNAs (miRNAs)—small non‐coding RNAs that regulate gene expression through post‐transcriptional repression of target messenger RNAs—have emerged as key modulators of lung development and injury. Because individual miRNAs can regulate multiple transcripts simultaneously, alterations in miRNA expression may affect interconnected pathways controlling cell proliferation and differentiation, inflammation, antioxidant responses, apoptosis, extracellular matrix remodeling, angiogenesis and vascular development.

Importantly, miRNA dysregulation relevant to BPD may begin before birth, rather than arising exclusively as a consequence of postnatal lung injury. Alterations in the maternal–placental environment may modify fetal miRNA signaling during critical windows of lung development, potentially establishing a state of developmental susceptibility before premature delivery. The abrupt transition to extrauterine life, followed by exposure to hyperoxia, mechanical ventilation, inflammation and infection, may subsequently reshape these regulatory networks. miRNAs may therefore represent a molecular interface linking antenatal programming, postnatal injury and disrupted pulmonary development.

From this perspective, BPD may be viewed not simply as a consequence of prolonged neonatal respiratory support, but as the final phenotype of a fetal–neonatal biological continuum in which developmental susceptibility and postnatal exposures interact over time. Characterizing the temporal and compartment‐specific patterns of miRNA dysregulation across this continuum may provide insight into the molecular heterogeneity of BPD and help identify biological signatures associated with specific disease trajectories.

In this educational review, we examine current evidence on miRNA dysregulation across the fetal–neonatal continuum of BPD, integrating findings from the maternal–placental compartment, fetal and cord blood, neonatal circulation, respiratory samples and the developing lung. We focus on miRNA‐regulated pathways involved in placental dysfunction and fetal growth restriction, angiogenesis and VEGF signaling, oxidative stress and antioxidant responses and disrupted alveolar and pulmonary vascular development. Finally, we critically evaluate the potential of miRNAs as biomarkers for early risk stratification and molecular endotyping, as well as their emerging role as therapeutic targets in BPD.

## 2 MicroRNAs as Post‐Transcriptional Regulators of Lung Development and BPD Pathogenesis

MicroRNAs (miRNAs) are small non‐coding RNA molecules, approximately 20–24 nucleotides in length, that regulate gene expression primarily at the post‐transcriptional level. By binding predominantly to complementary sequences within the 3′ untranslated region (3′‐UTR) of target messenger RNAs, miRNAs promote mRNA degradation or inhibit translation [18]. Through these mechanisms, miRNAs participate in complex regulatory networks controlling fundamental biological processes, including cell proliferation, differentiation and apoptosis, metabolism, angiogenesis, extracellular matrix (ECM) remodeling, tissue repair and inflammatory and immune responses [19, 20]. Although miRNAs are frequently considered within the broader framework of epigenetic regulation, their principal function is therefore to fine‐tune gene expression after transcription.

Lung development requires highly coordinated temporal and spatial regulation of gene expression. During the embryonic, pseudoglandular, canalicular, saccular and alveolar stages, dynamic changes in miRNA expression contribute to epithelial differentiation, airway branching, mesenchymal–epithelial interactions, pulmonary vascular development and ultimately alveolarization [21]. Mechanical stretch, hyperoxia, inflammation, infection and nutritional and metabolic disturbances may subsequently modify miRNA expression. Altered miRNA profiles have been reported both in experimental models of hyperoxia‐induced neonatal lung injury and in biological specimens from preterm infants with BPD, including peripheral blood, tracheal aspirates, extracellular vesicles and lung tissue [22]. Disruption of miRNA‐mediated regulatory networks may therefore represent an important molecular interface between developmental immaturity and postnatal injury.

Several miRNAs involved in lung growth and cellular homeostasis appear to be downregulated in association with impaired lung development. Among the best characterized is the miR‐17‐92 cluster, including miR‐17 and miR‐19b, which participates in pulmonary progenitor cell proliferation and epithelial morphogenesis. Experimental studies indicate that altered expression of this cluster can affect transforming growth factor‐β (TGF‐β) signaling, matrix metalloproteinases and extracellular matrix components including collagen Type III alpha 1 (COL3A1), thereby influencing distal lung development and alveolar septation [23, 24, 25].

Reduced expression of miR‐206, miR‐489, members of the let‐7 family and miR‐876‐3p has also been associated with pathways regulating cell‐cycle progression, differentiation and alveolar development [10, 20, 26, 27, 28, 29]. Altered expression of miRNAs involved in endothelial homeostasis and cellular survival—including miR‐150, miR‐126, miR‐342‐3p, miR‐342‐5p, miR‐425 and miR‐574‐3p—has been described under hyperoxic or inflammatory conditions [30, 31]. Among these, reduced miR‐425 expression has been linked to altered PI3K/Akt signaling and impaired cellular survival responses, potentially increasing susceptibility of the immature pulmonary epithelium to oxidative injury [32].

Conversely, adverse perinatal exposures may induce miRNAs capable of suppressing pathways required for normal alveolar and vascular development. miR‐34a is among the most extensively investigated examples. Its increased expression in experimental hyperoxia‐induced neonatal lung injury has been associated with disruption of pathways involved in alveolar development, angiogenesis and epithelial cell survival [33]. MiR‐421, through regulation of fibroblast growth factor 10 (FGF10)‐related signaling, has also been implicated in altered epithelial development and susceptibility to hyperoxic injury [34].

Changes in miR‐29a expression have been associated with dysregulated ECM turnover, potentially contributing to abnormal matrix remodeling rather than physiological alveolar septation [19]. More recently, transcriptomic analyses have identified increased miR‐219‐5p expression in association with severe BPD. Through regulation of platelet‐derived growth factor receptor‐α (PDGFR‐α) signaling, miR‐219‐5p may interfere with secondary septation and pulmonary microvascular development [35].

Taken together, these findings indicate that BPD‐associated miRNA dysregulation cannot be reduced to a simple pattern of protective miRNAs being downregulated and injurious miRNAs being upregulated. miRNA expression is highly context‐dependent and varies according to developmental stage, cell type, biological compartment, timing of sampling and exposure to hyperoxia, inflammation or other environmental stressors. Moreover, individual miRNAs may regulate multiple molecular targets, while individual signaling pathways may be simultaneously controlled by several miRNAs. Apparently divergent findings across experimental and clinical studies may therefore reflect different biological contexts rather than true inconsistencies.

A network‐based interpretation is consequently more appropriate. BPD appears to involve disruption of interconnected miRNA regulatory networks governing developmental, angiogenic, cytoprotective, inflammatory, apoptotic and ECM‐remodeling pathways. miRNA alterations may both reflect exposure to injurious stimuli and modify the biological response of the immature lung to those exposures. Thus, similar degrees of hyperoxia, inflammation or mechanical stress may have different developmental consequences depending on the underlying molecular regulatory environment.

Importantly, this regulatory vulnerability may be established before postnatal respiratory injury occurs. Alterations in the maternal–placental environment may influence fetal miRNA expression during critical developmental windows, potentially modifying pulmonary developmental trajectories and susceptibility to subsequent neonatal insults. This concept provides a biological link between fetal exposures and postnatal BPD and supports investigation of placental dysfunction with fetal growth restriction and chorioamnionitis/inflammation as components of the fetal origins of BPD [36, 37].

## 3 IUGR, Placental Dysfunction, Chorioamnionitis and MicroRNA Dysregulation: Fetal Origins of BPD

Increasing evidence suggests that susceptibility to bronchopulmonary dysplasia may be established before birth, rather than arising exclusively from postnatal exposures in the neonatal intensive care unit. Placental dysfunction and impaired fetal growth represent particularly important antenatal conditions capable of altering pulmonary development during critical developmental windows. Fetal growth restriction (FGR), frequently associated with placental insufficiency and chronic fetal hypoxemia, has emerged as an important antenatal risk factor for BPD. Consistently, clinical studies and meta‐analyses have shown that growth‐restricted preterm infants, as well as infants born small for gestational age (SGA), have an increased risk of BPD and severe respiratory morbidity compared with appropriately grown preterm infants [14, 38, 39, 40, 41].

Importantly, FGR and SGA describe related but distinct conditions. FGR refers to a pathological restriction of fetal growth resulting from failure to achieve the individual growth potential, frequently in the context of placental dysfunction, whereas SGA is defined according to birth weight relative to gestational age and does not necessarily imply pathological growth restriction. This distinction is relevant when investigating the fetal origins of BPD, because the underlying placental and fetal environment, rather than reduced body size per se, may represent the biological link between impaired fetal growth and abnormal lung development.

Experimental evidence indicates that placental insufficiency and FGR can interfere with pulmonary vascularization and distal lung maturation. Reduced angiogenic signaling, including alterations in vascular endothelial growth factor (VEGF) and VEGF receptor pathways, endothelial dysfunction, abnormal extracellular matrix remodeling and impaired alveolarization have been reported in experimental models of FGR [25, 38, 42]. These abnormalities resemble key features of contemporary BPD, in which disrupted pulmonary angiogenesis and simplified alveolar architecture are tightly interconnected.

Placental insufficiency also alters fetal oxygen and nutrient availability. Chronic fetal hypoxemia, oxidative stress, inflammation and mitochondrial dysfunction may modify developmental signaling before birth, potentially reducing the ability of the immature lung to adapt to the abrupt transition to extrauterine life. Rather than representing established lung injury, this state may be better conceptualized as a condition of developmental vulnerability, in which antenatal disturbances lower the threshold for injury from subsequent neonatal exposures such as hyperoxia, mechanical ventilation, infection and inflammation [25, 38, 42]. The biological consequences of postnatal respiratory exposures may therefore depend, at least in part, on developmental trajectories already established in utero.

Epigenetic and post‐transcriptional mechanisms may contribute to this fetal programming of pulmonary susceptibility. In particular, miRNAs provide a plausible mechanism through which placental dysfunction, hypoxia, inflammation and altered angiogenic signaling may influence fetal development [43, 44]. The placenta is highly enriched in miRNAs, and changes in placental miRNA expression have been associated with pregnancy complications characterized by abnormal placentation and impaired fetal growth.

Particular attention has focused on the chromosome 19 microRNA cluster (C19MC), one of the largest primate‐specific miRNA clusters. C19MC miRNAs are highly expressed in trophoblastic tissue and participate in regulatory networks involved in trophoblast differentiation, cellular proliferation, immune modulation, angiogenesis and adaptation to cellular stress [25, 42].

Altered expression of several C19MC‐associated miRNAs has been described in placentas from pregnancies complicated by FGR. Quantitative RT‐PCR studies have reported reduced expression of miR‐518b, miR‐1323, miR‐516b, miR‐515‐5p, miR‐520h, miR‐519d and miR‐526b compared with uncomplicated pregnancies [45, 46]. Because these miRNAs are involved in pathways regulating trophoblast function, vascular remodeling, endothelial homeostasis and cellular responses to hypoxia, their dysregulation may reflect impaired placental adaptation and altered maternal–fetal signaling [45, 47].

However, an important distinction should be emphasized. Although placental C19MC dysregulation is well documented in pregnancies complicated by impaired fetal growth, direct evidence linking specific C19MC alterations to subsequent BPD remains limited. It therefore remains unclear whether these miRNAs directly contribute to fetal lung programming, primarily serve as molecular markers of placental dysfunction or participate in both processes. Establishing such a relationship will require studies linking placental miRNA profiles directly with neonatal respiratory outcomes.

The potential relevance of placental miRNA dysregulation to BPD becomes particularly compelling when considered in the context of pulmonary vascular development. Angiogenesis and alveolarization are tightly coupled during fetal and early postnatal lung development, and disruption of endothelial signaling may therefore impair both vascular growth and secondary septation [45, 48]. Placental vascular disease may consequently influence the pulmonary vascular bed before birth and increase susceptibility to further endothelial injury after premature delivery.

These observations are consistent with the emerging concept that a subset of infants may develop BPD along a predominantly placental–vascular trajectory, associated with hypertensive disorders of pregnancy, placental vascular dysfunction, impaired fetal growth and subsequent abnormalities of the pulmonary circulation. Such infants may be particularly susceptible to pulmonary vascular disease and pulmonary hypertension. However, the molecular pathways defining this phenotype remain incompletely characterized, and whether it represents a distinct biological BPD endotype requires further investigation. MiRNA profiling may provide an opportunity to better characterize this heterogeneity by linking antenatal placental biology with postnatal pulmonary phenotypes. Importantly, this would require longitudinal studies integrating maternal and placental miRNA profiles with fetal or cord blood measurements, neonatal circulating or respiratory miRNAs and subsequent pulmonary outcomes.

Taken together, current evidence supports a model in which placental dysfunction may establish biological susceptibility to BPD before birth. Impaired placental vascularization, altered oxygen and nutrient delivery, oxidative and inflammatory stress and dysregulated placental miRNA networks may modify fetal developmental trajectories. Premature birth then superimposes additional environmental stressors on this vulnerable biological background.

Accordingly, BPD may be viewed as the result of a fetal–neonatal continuum of molecular dysregulation, rather than exclusively as a consequence of postnatal lung injury. Characterizing maternal, placental, fetal and neonatal miRNA profiles longitudinally may help distinguish different biological endotypes of BPD and identify infants at risk before conventional clinical manifestations become apparent.

Among the pathways potentially linking this antenatal susceptibility to postnatal lung development, angiogenic signaling is particularly relevant. The interaction between miRNAs and the VEGF/VEGFR axis, and its potential contribution to disrupted pulmonary vascular and alveolar development in BPD, is therefore considered in the following section.

## 4 MiRNA‐Mediated Regulation of Angiogenesis and VEGF Signaling in BPD

Disruption of pulmonary angiogenesis is a central feature of contemporary bronchopulmonary dysplasia, in which impaired vascular growth occurs in parallel with arrested alveolar development. MicroRNAs (miRNAs) have emerged as important regulators of this angiogenic–alveolar coupling by modulating vascular endothelial growth factor (VEGF) expression and multiple components of VEGF‐related signaling pathways [49].

VEGF is a critical regulator of fetal organogenesis and plays a fundamental role in pulmonary vascular and alveolar development. Beyond its established function in endothelial proliferation, migration and vascular growth, VEGF signaling contributes to epithelial–endothelial crosstalk during distal lung development. Experimental studies indicate that VEGF supports alveolar epithelial maturation, including Type II pneumocyte differentiation and surfactant‐related functions, whereas disruption of VEGF signaling results in endothelial and epithelial apoptosis, impaired pulmonary vascular growth, enlarged distal air spaces, defective alveolarization and altered lung mechanics [10, 50, 51, 52].

Clinical observations support the potential relevance of early VEGF dysregulation in BPD. Mariduena et al. reported lower circulating VEGF concentrations during the first week of life in preterm infants who subsequently developed BPD [53]. Although circulating VEGF concentrations may not directly reflect local pulmonary VEGF activity, these findings suggest that impaired angiogenic signaling may be detectable early in infants at increased risk of disrupted lung development. Importantly, VEGF expression is regulated at multiple levels, including post‐transcriptional control by miRNAs. Dysregulation of specific miRNAs following premature birth may therefore provide one mechanism through which hyperoxia, inflammation and other neonatal exposures interfere with pulmonary angiogenesis.

Among the miRNAs implicated in this pathway, miR‐451 has been investigated in experimental BPD. In a murine model of BPD, miR‐451 inhibition increased macrophage migration inhibitory factor (MIF) expression and attenuated the cardiopulmonary phenotype. Moreover, inhibition of miR‐451 was associated with restoration of VEGF‐A expression and modulation of additional angiogenic mediators, including angiopoietin‐1 (Ang1), angiopoietin‐2 (Ang2) and their receptor Tie2, accompanied by improved alveolar and vascular development [54]. These observations support a role for miR‐451 in the regulation of angiogenic responses during hyperoxic lung injury. The capacity of miR‐451 to influence VEGF‐related pathways is also supported by studies outside the neonatal lung. In oncological models, miR‐451 has been shown to modulate angiogenesis through pathways involving IL‐6R/STAT3 and mTOR/HIF‐1α/VEGF signaling [55, 56]. These observations support a potential role for miR‐451 in pathways relevant to vascular and pulmonary adaptation; however, direct evidence connecting miR‐451 with VEGF‐mediated angiogenesis in the developing human lung remains limited.

miR‐16 represents another potential regulator of VEGF expression. Experimental studies have demonstrated that miR‐16 can bind the 3′ untranslated region (3′‐UTR) of VEGF mRNA, reducing VEGF expression and suppressing angiogenic activity [57, 58]. This mechanism is potentially relevant to BPD, in which adequate VEGF signaling is required for coordinated pulmonary vascular and alveolar development. However, direct evidence defining the role of miR‐16 in human BPD remains limited. Its contribution should therefore currently be regarded as biologically plausible rather than established.

More direct evidence in the context of BPD has been reported for miR‐203a‐3p. Increased miR‐203a‐3p expression has been described in peripheral blood samples from infants with BPD, and VEGFA has been identified as one of its molecular targets [59]. Complementary experiments in hyperoxia‐exposed neonatal rats and alveolar epithelial cells demonstrated that increased miR‐203a‐3p expression was associated with reduced VEGFA signaling and increased apoptosis. Conversely, inhibition of miR‐203a‐3p restored VEGFA expression, modulated downstream ERK and PI3K signaling and attenuated hyperoxia‐induced lung injury [59]. These findings are particularly relevant because they connect a circulating miRNA identified in infants with BPD with a biologically plausible molecular target and complementary mechanistic evidence in experimental models.

The influence of miRNAs on pulmonary angiogenesis extends beyond direct regulation of VEGF expression. miR‐30a, for example, modulates endothelial responses to angiogenic signaling through regulation of Delta‐like ligand 4 (DLL4), a key component of the Notch pathway involved in endothelial tip‐cell selection and vascular sprouting. Under hyperoxic conditions, reduced miR‐30a expression has been associated with dysregulated DLL4/Notch signaling and abnormal pulmonary vascular development [60, 61]. miRNA dysregulation may therefore impair angiogenesis not only by altering VEGF availability but also by disrupting the endothelial signaling networks required to translate angiogenic stimuli into coordinated vascular growth.

All these findings support a model in which miRNAs regulate pulmonary angiogenesis at multiple levels. Some, such as miR‐16 and miR‐203a‐3p, can directly regulate VEGF expression, whereas others, including miR‐30a and potentially miR‐451, influence broader signaling networks involving DLL4/Notch, HIF‐1α, PI3K/Akt, STAT3 and other pathways relevant to endothelial function. Dysregulation of these networks may disrupt endothelial–epithelial crosstalk and thereby contribute simultaneously to impaired vascular growth and arrested alveolarization, two defining features of contemporary BPD (Figure 1) and its different prevalent phenotypes (‘lung parenchymal disease’, ‘pulmonary vascular disease’, ‘airway disease’ and mixed patterns) [62].

**FIGURE 1:**
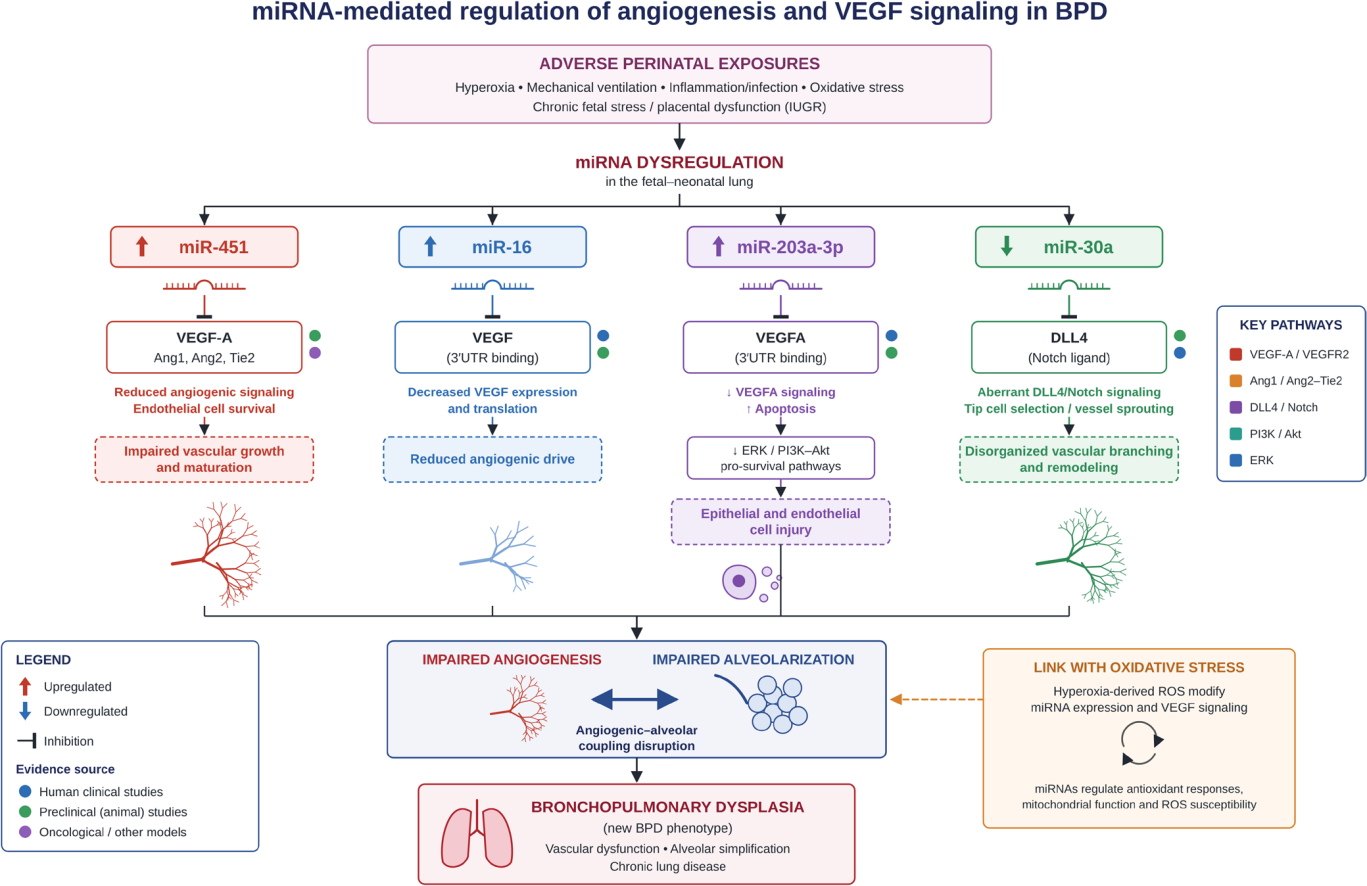
Schematic representation of the main microRNA (miRNA)‐regulated pathways linking prematurity‐related insults to impaired pulmonary angiogenesis and alveolar development in bronchopulmonary dysplasia (BPD). Hyperoxia, inflammation and mechanical ventilation may alter miRNA expression and disrupt vascular endothelial growth factor (VEGF)‐dependent signaling. miR‐16 and miR‐203a‐3p can directly regulate VEGF/VEGFA expression, whereas miR‐30a modulates the DLL4/Notch pathway involved in endothelial tip‐cell selection and vascular sprouting. miR‐451 participates in a broader regulatory network involving macrophage migration inhibitory factor (MIF) and vascular growth factors, including VEGF‐A and the Ang/Tie2 system. Dysregulation of these interconnected pathways may impair endothelial–epithelial crosstalk, pulmonary vascular growth and alveolarization, ultimately contributing to BPD. Solid lines indicate experimentally supported interactions, whereas dashed lines indicate proposed or indirect regulatory relationships requiring further validation in the developing human lung. Ang, angiopoietin; BPD, bronchopulmonary dysplasia; DLL4, delta‐like ligand 4; MIF, macrophage migration inhibitory factor; miRNA, microRNA; VEGF, vascular endothelial growth factor.

However, the strength of evidence differs substantially among individual miRNAs. Direct mechanistic evidence is available for selected miRNA–target interactions, whereas for others the proposed relationship with BPD derives predominantly from experimental hyperoxia models or from mechanistic studies conducted in other disease settings. Human neonatal evidence remains comparatively limited. Future longitudinal studies integrating miRNA profiles with angiogenic biomarkers and respiratory outcomes will therefore be essential to distinguish molecular alterations that precede BPD from those that primarily reflect established lung injury.

Angiogenic signaling and oxidative stress should not be considered independent pathogenic pathways. Hyperoxia‐derived reactive oxygen species can alter VEGF signaling and miRNA expression, whereas miRNAs can in turn regulate mitochondrial function, cellular antioxidant responses and susceptibility to oxidative injury. This reciprocal interaction provides a mechanistic link between vascular growth failure and the oxidative environment of the premature lung and introduces the role of miRNA networks in neonatal redox homeostasis.

## 5 MicroRNA Networks and Oxidative Stress in the Preterm Lung

Oxidative stress is a major contributor to lung injury and disrupted development in bronchopulmonary dysplasia (BPD) [63]. Preterm infants are particularly vulnerable to oxidative injury because antioxidant defense systems mature predominantly during late gestation. Premature birth interrupts this developmental process and simultaneously exposes the immature lung to a rapid increase in oxygen tension, often compounded by supplemental oxygen, mechanical ventilation, inflammation and infection. The resulting imbalance between reactive oxygen species (ROS) generation and antioxidant capacity creates a highly vulnerable redox environment during a critical period of alveolar and vascular development [64].

Increasing evidence indicates that oxidative stress and microRNAs are connected through a bidirectional regulatory network. Increased oxygen tension and ROS accumulation can modify miRNA transcription, processing and stability, thereby reshaping the cellular miRNA profile. Conversely, redox‐responsive miRNAs can regulate genes controlling mitochondrial function, antioxidant defenses, apoptosis, inflammation and ROS generation. Thus, miRNA dysregulation may be both a consequence of oxidative stress and an active determinant of the cellular response to oxidative injury. This reciprocal relationship is particularly relevant to the premature lung, where even relatively small changes in redox regulation may influence whether epithelial and endothelial cells successfully adapt to extrauterine oxygen exposure or progress toward apoptosis, inflammation and developmental arrest.

miR‐34a represents one of the best‐characterized hyperoxia‐responsive miRNAs implicated in BPD. Increased miR‐34a expression has been reported in experimental neonatal hyperoxia and in respiratory samples from preterm infants developing BPD [33, 65] Experimental evidence indicates that miR‐34a modulates pathways involved in epithelial cell survival, senescence and apoptosis, thereby potentially amplifying the consequences of oxidative injury in the developing lung. Similarly, miR‐195 is induced by hyperoxic exposure in experimental neonatal lung injury. Increased miR‐195 expression has been associated with intracellular ROS accumulation, apoptosis and impaired lung development, whereas experimental inhibition of miR‐195 reduced oxidative injury and preserved alveolar and microvascular architecture [66]. These findings illustrate how ROS‐induced changes in miRNA expression may translate an environmental exposure into a sustained molecular response capable of influencing lung development.

Hyperoxia may also suppress miRNAs involved in limiting oxidative injury. miR‐3202 has been implicated in this mechanism through regulation of Recombination Activating Gene 1 (RAG1). Reduced miR‐3202 expression has been described under experimental hyperoxic conditions, whereas restoration of miR‐3202 decreased RAG1 expression, intracellular ROS accumulation and epithelial apoptosis [67]. The miR‐3202/RAG1 axis therefore provides an example of how altered miRNA signaling may contribute to amplification of oxidative injury. A potentially different adaptive pattern has been described for miR‐574‐3p and adrenomedullin (ADM). ADM is a vasoactive peptide with antioxidant, anti‐inflammatory and endothelial‐protective properties that may contribute to pulmonary adaptation to oxidative stress. Reduced miR‐574‐3p expression has been reported in association with BPD, and ADM has been identified as one of its targets [30]. Because reduced miR‐574‐3p could relieve post‐transcriptional repression of ADM, this expression pattern has been proposed to represent an adaptive response aimed at enhancing endogenous cytoprotection. Whether this mechanism provides effective protection in the human preterm lung remains to be established.

An especially important intersection between miRNA signaling and redox homeostasis involves nuclear factor erythroid 2‐related factor 2 (NRF2), a master transcriptional regulator of cellular antioxidant defense. Under basal conditions, NRF2 is maintained at low intracellular levels largely through KEAP1‐mediated ubiquitination and proteasomal degradation. In response to oxidative stress, disruption of KEAP1‐dependent NRF2 degradation allows NRF2 accumulation and nuclear translocation, where it binds antioxidant response elements (AREs) and promotes transcription of cytoprotective genes involved in detoxification, glutathione metabolism and ROS neutralization, including heme oxygenase‐1 (HO‐1), NAD(P)H quinone oxidoreductase 1 (NQO1) and enzymes involved in glutathione synthesis and recycling.

Several miRNAs can modulate components of this antioxidant pathway. miR‐144 is of particular interest because NRF2 has been experimentally validated as a direct target of miR‐144 in redox‐sensitive cellular systems. Increased miR‐144 expression can reduce NRF2 abundance and attenuate NRF2‐dependent antioxidant responses, thereby decreasing cellular tolerance to oxidative stress [68, 69, 70]. Conversely, reduced miR‐144 expression could theoretically relieve NRF2 repression and facilitate antioxidant adaptation. This relationship provides an example of how changes in miRNA expression may not necessarily represent injury themselves but may instead reflect an attempt to restore redox homeostasis. In the context of the preterm lung, the biological significance of miR‐144 may therefore depend on both the direction and timing of its expression relative to oxidative exposure.

miR‐34a may influence antioxidant responses through related pathways, including regulation of SIRT1. SIRT1 participates in cellular stress responses and can modulate NRF2 activity through deacetylation‐dependent mechanisms [19, 68, 69, 70, 71]. Dysregulation of miRNAs affecting either NRF2 directly or upstream regulators such as SIRT1 may therefore impair the ability of the immature lung to mount an adequate antioxidant response to hyperoxic exposure. However, evidence supporting individual components of the miRNA–SIRT1–NRF2 network derives from different experimental systems, and direct demonstration of the complete regulatory axis in human BPD remains limited. Its role in the preterm lung should therefore currently be considered a biologically compelling mechanistic hypothesis requiring further validation.

Taken together, current evidence suggests that miRNAs participate in oxidative lung injury through at least three complementary mechanisms: ROS‐responsive miRNAs, whose expression is altered by hyperoxia and oxidative stress; redox‐regulating miRNAs, which directly influence ROS generation or antioxidant pathways; and adaptive miRNA responses, which may attempt to restore cellular homeostasis under sustained oxidative challenge. These categories should not be regarded as mutually exclusive. The biological effect of an individual miRNA is likely to depend on developmental stage, cellular source, timing, magnitude of oxidative exposure and the broader molecular environment.

Rather than operating as a linear pathway, oxidative stress and miRNA dysregulation may therefore form a self‐reinforcing feedback network (Figure 2). Oxidative stress modifies miRNA expression; altered miRNA profiles can subsequently weaken antioxidant defenses or enhance ROS‐generating pathways, and the resulting redox imbalance may further reshape miRNA expression. Persistence of this loop in the developmentally immature lung may contribute to epithelial and endothelial dysfunction, apoptosis, inflammation, impaired angiogenesis and ultimately arrested alveolarization.

**FIGURE 2:**
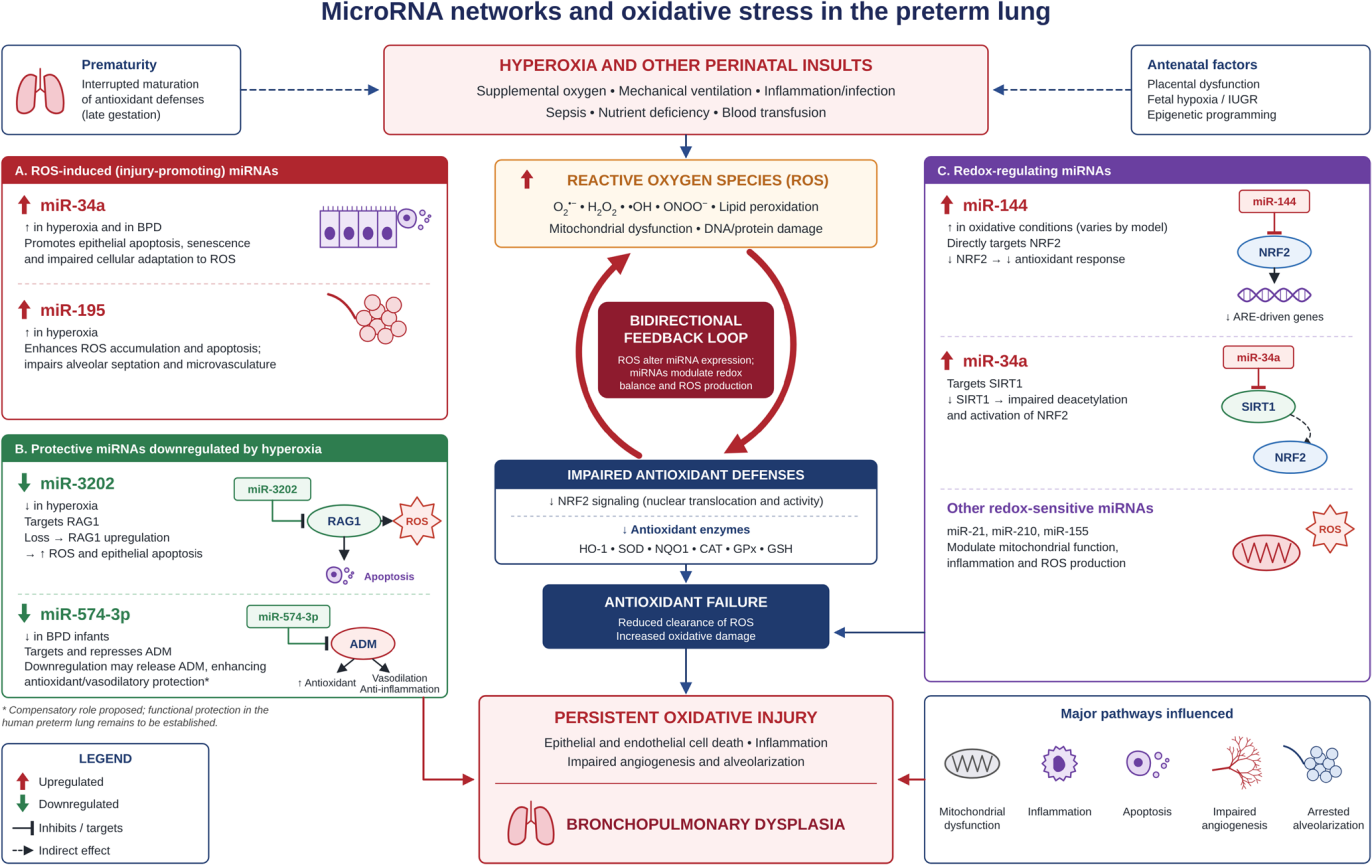
Schematic representation of the bidirectional relationship between oxidative stress and miRNA dysregulation in the developing preterm lung. Prematurity, antenatal vulnerability, hyperoxia, mechanical ventilation, inflammation and infection promote reactive oxygen species (ROS) generation in the setting of immature antioxidant defenses. Oxidative stress can modify miRNA expression, whereas redox‐responsive miRNAs may in turn regulate ROS generation, mitochondrial function, apoptosis and endogenous antioxidant pathways, creating a potential self‐reinforcing redox–miRNA feedback loop. Hyperoxia‐responsive miRNAs, including miR‐34a and miR‐195, have been associated with oxidative injury and impaired lung development, whereas miR‐3202 and miR‐574‐3p participate in potentially cytoprotective or adaptive pathways. miR‐144 provides an important link with the NRF2 antioxidant pathway, as NRF2 is a validated target of miR‐144 in redox‐sensitive systems; altered miR‐144 expression may therefore influence NRF2‐dependent antioxidant responses. Persistence of redox–miRNA dysregulation may contribute to mitochondrial dysfunction, inflammation, epithelial and endothelial injury, impaired angiogenesis, arrested alveolarization and ultimately BPD. ADM, adrenomedullin; ARE, antioxidant response element; BPD, bronchopulmonary dysplasia; HO‐1, heme oxygenase‐1; KEAP1, Kelch‐like ECH‐associated protein 1; miRNA, microRNA; NQO1, NAD(P)H quinone oxidoreductase 1; NRF2, nuclear factor erythroid 2‐related factor 2; RAG1, recombination activating gene 1; ROS, reactive oxygen species; SIRT1, sirtuin 1.

This model also provides a molecular connection between antenatal susceptibility and postnatal injury. Placental dysfunction, fetal hypoxia and altered intrauterine redox signaling may influence miRNA networks before birth, whereas premature exposure to hyperoxia, inflammation and mechanical ventilation may further remodel these networks after delivery. A dynamic redox–miRNA signature may therefore represent one of the molecular mechanisms linking the fetal and neonatal phases of BPD pathogenesis.

The growing understanding of microRNA (miRNA)‐mediated regulation of lung development, angiogenesis, inflammation and oxidative stress has generated considerable interest in their potential clinical application in bronchopulmonary dysplasia (BPD). Their translational relevance is twofold. First, altered miRNA profiles may provide early molecular signatures of disease susceptibility before BPD becomes clinically established. Second, because miRNAs actively regulate pathways involved in lung injury and repair, selected miRNAs may represent potential targets for therapeutic modulation. However, substantial methodological and biological challenges remain before either application can be translated into neonatal clinical practice.

### 5.1 Circulating miRNAs as Early Biomarkers of BPD

An ideal biomarker for BPD should be detectable early, require minimally invasive sampling, provide information beyond established clinical risk factors and reflect biological processes involved in disease development rather than established lung injury alone. miRNAs possess several characteristics that make them attractive candidates. They are detectable in multiple biological fluids, are relatively stable because of their association with proteins or encapsulation within extracellular vesicles, and can be quantified using highly sensitive molecular techniques. Moreover, their expression may change rapidly in response to hypoxia, hyperoxia, inflammation, oxidative stress and altered angiogenic signaling.

These properties are particularly relevant because current clinical definitions identify BPD relatively late in the disease trajectory, generally according to respiratory support requirements at 36 weeks' postmenstrual age. By this time, the biological processes leading to arrested alveolar and vascular development have been active for weeks. The potential value of miRNA profiling therefore lies primarily in identifying molecular trajectories toward BPD during an earlier and potentially modifiable phase of disease development.

Several miRNAs associated with BPD‐related pathways have been detected in peripheral blood, cord blood, tracheal aspirates, lung tissue and extracellular vesicles. However, individual miRNAs are unlikely to provide sufficient specificity for a multifactorial and heterogeneous developmental disorder such as BPD. A more promising strategy may be the identification of multimarker miRNA signatures reflecting complementary biological processes, including inflammation, oxidative stress, angiogenesis, epithelial injury and impaired alveolarization.

### 5.2 Biological Compartment and Timing: Where and When Should miRNAs be Measured?

The fetal–neonatal continuum discussed throughout this review raises an important translational question: not only which miRNA should be measured, but also where and when it should be measured.

Placental miRNAs may provide information on antenatal programming and placental dysfunction, potentially identifying biological susceptibility before neonatal respiratory injury occurs. Cord blood represents an attractive interface between fetal and neonatal life and can be obtained without additional blood sampling from the infant. However, cord blood miRNA profiles may reflect contributions from fetal, placental and maternal compartments and therefore cannot automatically be interpreted as lung‐specific.

Peripheral serum and plasma allow repeated longitudinal assessment and may therefore be particularly useful for characterizing dynamic changes during postnatal adaptation. Nevertheless, circulating miRNA concentrations are low and are influenced by hemolysis, platelet contamination, sample processing and other pre‐analytical factors.

Respiratory samples, particularly tracheal aspirates, may provide molecular information more closely related to events occurring within the lung. Their major limitation is that they are generally available only from intubated infants, introducing potential selection bias toward the most critically ill population. Extracellular vesicles offer additional opportunities because they protect miRNAs from degradation and participate in intercellular signaling; however, methods for their isolation, characterization and miRNA quantification remain insufficiently standardized for routine clinical use.

The biological significance of a miRNA signal must therefore be interpreted according to both its anatomical compartment and developmental timing. A placental miRNA measured at delivery, a circulating miRNA measured during the first days after birth and a tracheal aspirate miRNA obtained after prolonged respiratory support may represent fundamentally different stages of the disease process.

### 5.3 From Association to Prediction: Major Barriers to Clinical Implementation

Despite the rapidly expanding literature, no miRNA‐based biomarker has yet been incorporated into routine BPD risk assessment. Several factors contribute to this translational gap.

Most available studies include relatively small cohorts, frequently from single centers, with substantial heterogeneity in gestational age, respiratory management, sampling time points and BPD definitions. Methodological variability further limits comparison among studies. Serum, plasma, whole blood, tracheal aspirates and extracellular vesicles are not biologically interchangeable, and differences in sample collection, centrifugation, storage, RNA extraction, reverse transcription analytical platforms and normalization strategies can substantially influence measured miRNA expression.

Normalization represents a particularly important challenge for circulating miRNAs. The choice between endogenous reference miRNAs, exogenous spike‐in controls and global normalization approaches may affect both the magnitude and apparent direction of differential expression. Hemolysis is another relevant confounder because erythrocytes and platelets contain abundant miRNAs, and even limited contamination may substantially alter circulating profiles.

Biological confounding is equally important. Infants who develop BPD are generally born at lower gestational ages and birth weights and experience greater exposure to supplemental oxygen, mechanical ventilation, inflammation and infection. Consequently, a miRNA associated with BPD may reflect developmental immaturity or treatment exposure rather than disease‐specific biology. Furthermore, miRNA expression is highly context‐dependent and may vary according to cell type, developmental stage, biological compartment and timing of sampling, potentially explaining apparently conflicting directions of dysregulation across studies.

The major challenge is therefore not simply to identify miRNAs that differ between infants with and without BPD, but to demonstrate that miRNA profiles provide clinically meaningful information beyond established risk factors. Future studies should include adequately powered prospective cohorts, standardized sampling and analytical protocols, multivariable adjustment for major clinical confounders, and independent validation populations. Evaluation of discrimination, calibration and incremental predictive value will be essential before miRNA signatures can be considered clinically useful.

Longitudinal approaches may be particularly informative. Rather than relying on a single measurement, changes in miRNA expression from fetal life through early postnatal adaptation may identify trajectories associated with successful pulmonary adaptation or progression toward BPD.

### 5.4 miRNAs as Therapeutic Targets: From Molecular Modulation to Lung‐Specific Delivery

The functional involvement of miRNAs in BPD‐related pathways also raises the possibility of therapeutic modulation. Two complementary strategies are being explored experimentally: miRNA mimics, which aim to restore miRNAs whose protective functions are reduced during lung injury, and anti‐miRs or antagomiRs, which inhibit miRNAs whose increased activity contributes to pathological signaling.

Preclinical studies provide proof of principle that selected miRNA pathways are functionally modifiable in experimental BPD. Targeting the miR‐34a/PDGFRα axis partially restored alveologenesis in experimental BPD, supporting a functional contribution of miR‐34a dysregulation to arrested lung development [33]. More recent experimental evidence further supports the involvement of miR‐34a in hyperoxia‐induced neonatal lung injury [72].

Similarly, inhibition of miR‐451 in a murine model of BPD preserved MIF expression and attenuated the cardiopulmonary phenotype, with improved lung morphometry, vascular density and pulmonary vascular remodeling. miR‐451 inhibition was also associated with increased expression of VEGF‐A, angiopoietin‐1, angiopoietin‐2 and Tie2, further supporting a functional relationship between miRNA regulation and pulmonary vascular development [54].

Additional therapeutic opportunities may arise from miRNAs involved in endothelial dysfunction. In neonatal hyperoxic lung injury, macrophage‐derived extracellular vesicle‐associated miR‐23a‐3p impaired endothelial progenitor cell maintenance and angiogenic capacity [73]. Although direct therapeutic inhibition was not evaluated, these findings identify miR‐23a‐3p signaling as a potentially modifiable pathway relevant to pulmonary vascular development.

The transition from experimental target identification to clinical therapy, however, requires effective and tissue‐specific delivery. Extracellular vesicles are particularly attractive because they function as endogenous carriers of miRNAs and may potentially be engineered to deliver regulatory molecules to selected cell populations [74]. Lipid nanoparticles and other nanocarriers represent additional possibilities.

The respiratory tract may offer a particular advantage because local administration could maximize pulmonary exposure while limiting systemic effects. Exogenous surfactant represents an intriguing potential carrier in preterm infants because it is already delivered directly to the immature lung as part of routine neonatal care. Although surfactant has not yet been established as a vehicle for miRNA therapy in BPD, experimental studies in preterm rabbits have demonstrated the feasibility of combining surfactant with pharmacologically active molecules to attenuate ventilation‐ and hyperoxia‐induced lung injury, providing proof of concept for surfactant‐assisted pulmonary drug delivery [75].

Nevertheless, the ability of individual miRNAs to regulate multiple molecular targets represents both a therapeutic opportunity and a substantial potential risk. A single miRNA may influence numerous transcripts and interconnected pathways across several organs. Therapeutic manipulation could therefore produce unintended developmental effects, particularly in extremely preterm infants, in whom the lung, brain, retina, intestine and other organs are simultaneously undergoing rapid maturation.

Successful translation will require precise identification of disease‐relevant targets, cell‐specific delivery, appropriate dose and timing, characterization of biodistribution and duration of effect, and rigorous evaluation of immunogenicity and off‐target effects. Developmental timing is particularly important because the same miRNA pathway may have different functions during canalicular, saccular and alveolar stages of lung development. miRNA‐based therapy for BPD should therefore currently be regarded as a promising preclinical strategy rather than an immediately translatable intervention.

### 5.5 Toward a Longitudinal Molecular Signature of BPD

The future clinical relevance of miRNAs in BPD may ultimately depend on moving beyond the search for a single disease‐specific miRNA. BPD is biologically heterogeneous, and different infants may reach a similar clinical phenotype through predominantly inflammatory, vascular, oxidative, infectious or placental pathways. miRNA profiles may therefore be particularly valuable as components of molecular endotyping, helping to identify distinct biological trajectories within the broader clinical diagnosis of BPD.

This concept is consistent with the fetal–neonatal continuum proposed in this review. Placental miRNA dysregulation may reflect antenatal susceptibility; cord blood profiles may capture the molecular state at the fetal‐to‐neonatal transition; serial circulating miRNAs may characterize postnatal adaptation to oxidative and inflammatory stress; and respiratory or extracellular‐vesicle miRNAs may provide information more directly related to evolving pulmonary injury.

Future studies should therefore integrate longitudinal, multi‐compartment miRNA profiling with clinical phenotyping and complementary omics approaches, including genomics, transcriptomics, proteomics and metabolomics. Such integration may enable the transition from isolated biomarker associations toward dynamic molecular signatures capable of identifying BPD endotypes, improving early risk stratification and ultimately guiding individualized preventive or therapeutic strategies.

In this perspective, the translational promise of miRNAs lies not in identifying a single ‘BPD miRNA’, but in understanding how miRNA networks evolve across the fetal–neonatal continuum and interact with developmental and environmental exposures to determine individual susceptibility to chronic lung disease.

## 6 Conclusions and Future Perspectives

Bronchopulmonary dysplasia is a complex and heterogeneous developmental lung disorder arising from the interaction between antenatal susceptibility and postnatal injury. As highlighted throughout this review, microRNAs represent important post‐transcriptional regulators within the broader epigenetic landscape of BPD and may provide a molecular interface linking placental dysfunction, altered fetal development, premature transition to extrauterine life and subsequent lung injury.

Across this fetal–neonatal continuum, dysregulated miRNA networks intersect with multiple pathways central to BPD pathogenesis, including placental and fetal signaling, VEGF‐mediated angiogenesis, oxidative stress responses, inflammation, apoptosis and extracellular matrix remodeling. Placenta‐enriched miRNAs, including members of the C19MC cluster, may reflect antenatal developmental vulnerability, whereas postnatal changes in angiogenesis‐ and redox‐related miRNAs may influence the ability of the immature lung to adapt to hyperoxia, mechanical ventilation, inflammation and other neonatal exposures. Through these interconnected mechanisms, miRNA dysregulation may contribute to disruption of the physiological coupling between pulmonary vascular growth and alveolarization.

However, the direction of miRNA dysregulation should not be interpreted in isolation. miRNA expression is highly dependent on developmental stage, cellular source, biological compartment, timing of sampling and environmental exposure. The same miRNA may therefore show apparently divergent expression patterns across experimental models or clinical studies, and such differences do not necessarily represent biological inconsistency. Moreover, miRNA alterations associated with BPD may reflect developmental immaturity, environmental exposure, adaptive responses, established lung injury or a combination of these processes.

Current evidence should therefore be considered an initial framework rather than a definitive molecular map of BPD. Future research should move beyond cross‐sectional, single‐miRNA associations toward longitudinal and multi‐compartment profiling, tracking molecular changes from the maternal–placental environment and fetal circulation through the transition at birth and subsequent neonatal lung development. Integration with other regulatory layers—including DNA methylation, histone modifications, long non‐coding RNAs and chromatin remodeling—may further clarify how antenatal and postnatal exposures become biologically embedded during critical developmental windows.

Large, well‐characterized prospective cohorts will be essential to determine whether specific miRNA signatures identify biologically distinct BPD endotypes and provide clinically meaningful information beyond established risk factors. Standardization of biological sampling, pre‐analytical processing, RNA extraction, normalization strategies, analytical platforms and BPD definitions will be equally important to improve reproducibility and enable external validation.

The translational potential of miRNAs extends from early molecular phenotyping to therapeutic modulation. However, their clinical relevance will ultimately depend on demonstrating not only biological association but also temporal precedence, incremental predictive value, reproducibility, and, for therapeutic applications, developmental and long‐term safety. Ultimately, the future of miRNA research in BPD is unlikely to lie in the identification of a single ‘BPD miRNA’. A more informative approach will be to characterize dynamic miRNA networks across the fetal–neonatal continuum and determine how these networks interact with developmental stage and environmental exposures to generate distinct trajectories toward chronic lung disease. Integrating these molecular trajectories with clinical phenotyping and complementary omics approaches may enable a transition from a predominantly severity‐based definition of BPD toward a more biologically informed classification, with the long‐term goal of improving early risk stratification and developing individualized preventive and therapeutic strategies.

## Author Contributions

**Serafina Perrone:** conceptualization, investigation, funding acquisition, writing – original draft, writing – review and editing, visualization, validation. **Silvia Carloni:** investigation, writing – original draft, writing – review and editing, visualization. **Virginia Beretta:** writing – review and editing, visualization. **Vincenzo Raitano:** writing – review and editing, writing – original draft, visualization. **Pietro Leuratti:** writing – original draft, writing – review and editing. **Luca Buttarelli:** writing – original draft, writing – review and editing. **Elisa Caselli:** writing – original draft, writing – review and editing. **Sofia Gerevini:** writing – original draft, writing – review and editing. **Maria Cristina Albertini:** visualization, writing – review and editing. **Serena Benedetti:** visualization, writing – review and editing. **Francesco Nonnis‐Marzano:** visualization, writing – review and editing. **Gianfranco Maffei:** visualization, writing – review and editing. **Eloisa Gitto:** visualization, writing – review and editing. **Giovanni Vento:** visualization, writing – review and editing.

## Funding

This work was funded by a grant from PNRR‐MCNT‐2023‐12377464 ‘Ministero della Salute: new high‐risk profile for early detection and management starting from perinatal life’.

## Conflicts of Interest

The authors declare no conflicts of interest.

## Data Availability

Data sharing not applicable to this article as no datasets were generated or analysed during the current study.
